# Patient Satisfaction and Experience with Same-Day Discharge After Laparoscopic Roux-en-Y Gastric Bypass: A Mixed-Methods study

**DOI:** 10.1007/s11695-024-07264-8

**Published:** 2024-05-25

**Authors:** Suzanne C. Kleipool, Gijs J. A. Willinge, Elke G. E. Mathijssen, Kim A. G. J. Romijnders, Steve M. M. de Castro, Hendrik A. Marsman, Pim W. J. van Rutte, Ruben N. van Veen

**Affiliations:** 1 Department of Surgery, OLVG Hospital, Amsterdam, The Netherlands; 2 The Healthcare Innovation Center, University Medical Center Utrecht, Utrecht, The Netherlands; 3 Julius Center for Health Sciences and Primary Care, University Medical Center Utrecht, Utrecht, The Netherlands

**Keywords:** Bariatric surgery, Same-day discharge, Roux-en-Y gastric bypass, Patient satisfaction, Patient experience, Mixed-methods

## Abstract

**Introduction:**

Same-day discharge (SDD) after laparoscopic Roux-en-Y gastric bypass (RYGB) is a safe and effective healthcare pathway. However, there is limited understanding of the patient perspective on SDD. The aim of this study was to explore patient satisfaction and experience with SDD after RYGB.

**Methods:**

A mixed-methods study with a concurrent design was conducted in a Dutch teaching hospital, using questionnaires and interviews. Patients who underwent RYGB and were discharged on the day of the surgery completed four questionnaires of the BODY-Q (satisfaction with the surgeon, satisfaction with the medical team, satisfaction with the office staff, and satisfaction with information provision) ± 4 months postoperative. The results of the questionnaires were compared with pre-existing data from a cohort of patients who stayed overnight after surgery (i.e., control group). A subset of patients was individually interviewed for an in-depth understanding of the patient perspective on SDD.

**Results:**

In the questionnaires, median scores for the control group (*n* = 158) versus the present group of patients (*n* = 51) were as follows: 92/100 vs. 92/100 (*p* = 0.331) for the surgeon, 100/100 vs. 92/100 (*p* = 0.775) for the medical team, 100/100 vs. 100/100 (*p* = 0.616) for the office staff, and 90/100 vs. 73/100 (*p* = 0.015) for information provision. Interviews with 14 patients revealed seven themes, describing high satisfaction, along with several points of interest.

**Conclusions:**

Patient satisfaction with SDD after RYGB is high, although information provision regarding the day of surgery could be improved. However, not every medically eligible patient might be suitable for this healthcare pathway, as responsibilities are shifted.

**Graphical Abstract:**

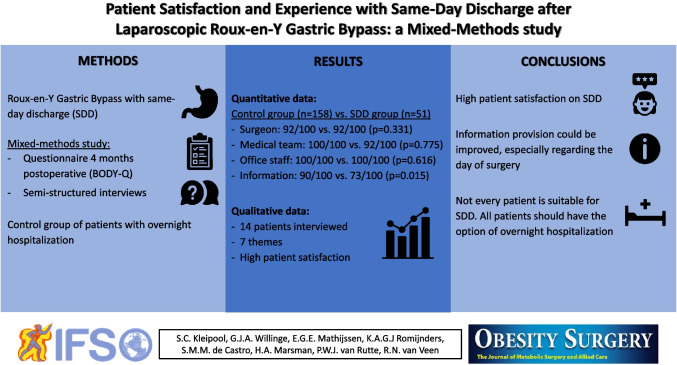

## Introduction

Bariatric surgery with same-day discharge (SDD) is an innovative and effective healthcare pathway. This SDD pathway has demonstrated feasibility and safety, provided that several criteria are followed [1–7]. These criteria include strict patient selection, adherence to a standardized perioperative protocol, clear discharge criteria, effective patient expectation management and information provision, and the establishment of a safety net for the timely detection and management of potential early complications [1–7]. The SDD pathway has emerged from the reduction of admission time following the development of Enhanced Recovery After Bariatric Surgery (ERABS) [8]. This includes a multimodal strategy for analgesia, postoperative nausea and vomiting (PONV), and early postoperative mobilization [9]. The SDD pathway has the potential to alleviate the burden on hospital capacity, particularly with current challenges associated with local staff shortages and the recent COVID-19 pandemic, without compromising patient satisfaction [6, 10–12].

Patient satisfaction and experience are patient-centered measures of quality of care [13]. Larson et al. defined patient satisfaction as patients’ evaluation of received care relative to their expectations. Patient experience is defined as the interactions that patients have with the healthcare system [13]. Previous studies have indicated that patients express satisfaction with Enhanced Recovery after Surgery and SDD following various surgical procedures, including laparoscopic cholecystectomy, as well as diverse orthopedic and gynecological surgeries [12, 14–19]. Patient satisfaction with bariatric surgery with SDD has been investigated in a limited number of studies, mostly focusing on sleeve gastrectomy (SG), all reporting high satisfaction rates [20–22]. A study by Nijland et al. reported on the satisfaction rates of SDD after laparoscopic Roux-en-Y gastric bypass (RYGB) [11].

SDD after laparoscopic RYGB was implemented in 2020 in our hospital. Since its implementation, over 1000 patients have undergone successful RYGB with SDD. The aim of this study was to explore patient satisfaction and experience following laparoscopic RYGB with SDD.

## Methods

An explorative, descriptive mixed methods study with a concurrent design was conducted in a high volume bariatric center in the Netherlands between November 2022 and October 2023, using questionnaires and interviews. Quantitative and qualitative data were collected and analyzed separately and simultaneously. Each type of data was given equal weight (i.e., neither type of data dominates the other). The study was reported according to the Mixed-Methods Article Reporting Standards (MMARS). A waiver for ethical approval was provided by the research ethics committee (MREC NedMec). Local approval for this study was obtained from the hospital’s research board and the board of directors.

### Study Population

The study population consisted of patients aged between 18 and 65 years, who underwent primary laparoscopic RYGB with SDD. Proficiency in the Dutch language was a prerequisite for participation. The surgeries were conducted by experienced and certified bariatric surgeons in accordance with the international guidelines for bariatric and metabolic surgery [23]. Patients were treated according to a specific protocol for SDD, the details of which have been previously published [6, 7, 24]. Patients received a written document containing information on the study. Informed consent was obtained from patients prior to the initiation of any study-related activities. A convenience sampling technique was used for the quantitative research, selecting patients in order of the date of the surgery. A subset of patients was selected for the qualitative research, using a purposive sampling technique. The aim was to select patients of varying age and gender. The sample size was determined by the principle of data saturation [25].

### Data Collection

#### Quantitative Data

For the quantitative part of this study, the Dutch version of the BODY-Q questionnaire was used. This is a standardized and validated patient-reported outcome instrument, designed to evaluate outcomes for bariatric patients [26, 27]. It consisted of a collection of independently functioning scales, including a domain focusing on patient experience. This domain had four subscales that measure satisfaction with the surgeon, satisfaction with the medical team, satisfaction with the office staff, and satisfaction with the information provision. The response scale consisted of four-point scores: 1 (very dissatisfied/definitely disagree), 2 (somewhat dissatisfied/disagree), 3 (somewhat satisfied/agree), and 4 (very satisfied/definitely agree). Item responses for each scale were summed and converted to an equivalent Rasch transformed score that ranges from 0 (worst) to 100 (best) [27]. Patients were asked to complete these four online questionnaires approximately 4 months postoperative, utilizing the Castor Electronic Data Capture system [28]. Reminders were sent in cases of non-responders. The data were compared to the results of questionnaires collected at the same hospital approximately 4 months postoperative in 2018 and 2019 (i.e., control group). These patients all had a minimum of one night of hospitalization, as SDD after RYGB was not implemented at that time.

#### Qualitative Data

The interviews were one-on-one conversations and were conducted remotely via Microsoft Teams or telephone, 1–2 weeks postoperative. Three researchers (EM, KR, and WdL) with extensive experience with conducting interviews, no pre-existing relationship with the patients, and who were not involved with the treatment performed the interviews. An interview guide was used to semi-structure the conversations and ensure consistency between the interviewers (Appendix Table 4). Field notes were made during and directly after the interviews to collect contextual information. The interviews were audio-recorded and subsequently transcribed verbatim by a professional transcription service.

### Data Analysis

Quantitative data were presented as mean ± standard deviation (SD) or median (interquartile range), and categorical data were presented as counts and percentages. The normality of the variables was assessed through visual inspection of histograms and Q-Q plots. Normally distributed data were analyzed using an independent samples *t*-test, while the Mann–Whitney *U* test was used for non-normally distributed data. Categorical data were compared using a chi-square test. A *p*-value of *p* < 0.05 was considered significant. All statistical analysis were performed using SPSS version 22.0 (SPSS, Inc., Chicago, Ill.).

For the qualitative data analysis, inductive thematic analysis was applied to analyze the field notes and verbatim transcriptions. The qualitative analysis was guided by the analysis steps of Braun and Clarke [29]. Researcher triangulation was applied to minimize the risk of researcher bias, involving two researchers (EM and KR). NVivo version 1.7.1 facilitated the qualitative data analysis. Direct quotes of patients were used to illustrate the qualitative results.

## Results

There were 67 patients who consented to participate in the present study, all of whom underwent RYGB with successful SDD. Among these patients, 51 completed the questionnaires (response rate of 76%) and 14 patients were also interviewed. The interviews lasted between 35 and 65 min. In the control group of patients with overnight hospitalization, 158 questionnaires were collected. Baseline characteristics are presented in Table 1. Overall, the patients who were treated with SDD were younger had lower body mass index (BMI) and exhibited significantly fewer comorbidities than the control group. This demographic profile aligns with the typical SDD population [6].

**Table 1:** Baseline characteristics

	Questionnaires control group (*n* = 158)	Questionnaires present study (*n* = 51)		Interviews present study (*n* = 14)
Age at surgery, years (mean, SD)	46 ± 9	38 ± 12	*p* < 0.001*	42 ± 11
Female (*n*, %)	133 (84.2)	49 (96.1)	*p* = 0.028^§^	13 (92.9)
Weight, kg (mean, SD)	121 ± 19	115 ± 13	*p* = 0.017*	119 ± 11
BMI, kg/m^2^ (mean, SD)	42 ± 5	41 ± 3	*p* = 0.012*	42 ± 3
Comorbidities (*n*, %)				
Hypertension	59 (37.3)	2 (3.9)	*p* < 0.001^§^	2 (14.3)
NIDDM	17 (10.8)	1 (2.0)	*p* = 0.051^§^	1 (7.1)
IDDM	12 (7.6)	0	*p* = 0.043^§^	0
Dyslipidemia	26 (16.5)	1 (2.0)	*p* = 0.007^§^	1 (7.1)

*BMI* body mass index; *IDDM* insulin dependent diabetes mellitus; *NIDDM* non-insulin dependent diabetes mellitus; *SD* standard deviation

^§^Chi-square

^*^Independent samples *t*-test

### Quantitative Data

For both groups, the quantitative data indicated that the majority of the patients were satisfied with the care received. The data from the control group of 158 patients showed a median satisfaction score of 92/100 for the surgeon, 100/100 for the medical team, 100/100 for the office staff, and 90/100 for information provision. The data from the present group of 51 patients revealed median satisfaction scores of 92/100 for the surgeon, 92/100 for the medical team, 100/100 for the office staff, and 73/100 for information provision. A single statistically significant difference was observed between the quantitative results of the control group and the SDD group, namely in the median satisfaction score for information provision (90/100 vs. 73/100, *p* = 0.015). When examining the individual questions within the questionnaires of the SDD group, there were no questions that received an overall score lower than “somewhat satisfied” or “somewhat agree.” The results are presented in Table 2.

**Table 2:** Outcomes quantitative research

	Control group (median, IQR) *n* = 158	Present study (median, IQR) *n* = 51	
Surgeon	92 (58–100)	92 (77–100)	*p* = 0.331^¥^
Medical team	100 (74–100)	92 (78–100)	*p* = 0.775^¥^
Office staff	100 (75–100)	100 (75–100)	*p* = 0.616^¥^
Information provision	90 (69–100)	73 (55–100)	***p* = 0.015**^**¥**^

*IQR* interquartile range

^¥^Mann Whitney *U*

### Qualitative Data

**Fig. 1:**
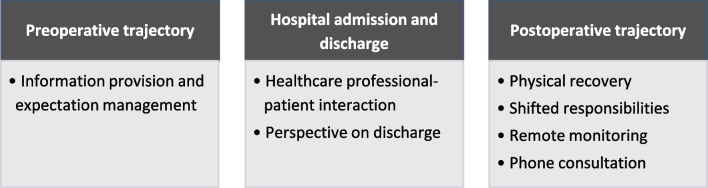
Categorization of themes that described patient satisfaction and experience

The qualitative analysis revealed seven themes that described patient satisfaction and experience with SDD after RYGB. The themes were categorized according to the following components of treatment: preoperative trajectory, hospital admission and discharge, and the postoperative trajectory (Fig. 1). A selection of direct quotes of patients are shown in Table 3.

**Table 3:** Direct quotes of patients

Theme	#	Quote
Information provision and expectation management	1	That you didn’t really know exactly how everything would unfold. What time you would be operated on. Which medications you would be taking home. All those things
Healthcare professional-patient interaction	2	I felt very comfortable the whole time, so I was really happy about that. Yeah. Surgery is still a big deal, and it remains somewhat nerve-wracking, but the healthcare staff really helped a lot. That is very reassuring
	3	So yeah, I can’t say anything else than that I’ve experienced it as very nice. Not that undergoing surgery is very pleasant, but the way they treat you is
Perspective on discharge	4	I was still very groggy, so I would occasionally drift off, but it was very clear for my husband. He was able to tell me everything the next day. They provide very clear information, and I know that, but I just couldn’t follow everything because I kept drifting off at times
	5	If I had wanted to, it would have been allowed. If it felt better for me, you know. Then I could have stayed. No, I didn’t have that conversation. It was more like, you go home at 4 o’clock. But I know from others that you were allowed to stay if you didn’t feel well or something. So I didn’t discuss it with them personally. No
	6	I didn’t have any problems at all, so yeah, it felt fine to me. If I had had any doubts, I would have discussed it and said, “Hey, I want to stay.” But it felt fine to me
Physical recovery	7	You see, I think the general principle is that people prefer to sleep in their own bed. And that applies to me as well
	8	I just wanted to go home. I think, no, I know for myself, I feel best at home
Shifted responsibilities	9	I think… You can’t expect people to always assess every situation correctly. And that is really the responsibility of the healthcare provider
	10	You get quite a few responsibilities as a partner because someone has to do the checks, measure oxygen, heart rate, and temperature. Someone has to be woken up at 3 in the morning to see if they’re “with it” because otherwise, they might die from internal bleeding. Well, I think it’s quite a lot. You’re placing quite a responsibility on someone who is not medically trained
Remote monitoring	11	You know, it’s not rocket science. It’s taking temperature, recording it, the oxygen, and what was it? I think the heart rate, something like that
	12	Well, it does provide a… Yeah. It does provide a certain kind of confidence. You think, “If I don’t trust it, at least I can measure.” And that’s all good. And they are… Yeah, like I said, fortunately simple devices that most people can understand, I think
Phone consultation	13	Yes, I actually experienced it as very reassuring. It’s a sign that they’re keeping an eye on you, that they haven’t forgotten about you even though you’re at home. It’s a form of aftercare, and I find that important. But if things aren’t going well, well… it’s not just up to you to initiate contact; they’re also monitoring it themselves
	14	Well, I might think things are going well, but that doesn’t necessarily mean they are. So, it’s nice to have someone else who has dealt with these situations more often confirm or deny it, as the case may be. But this time, it went very well

#### Preoperative Trajectory

##### Information Provision and Expectation Management

Generally, patients indicated that they received sufficient and adequate information to enable them to approach the surgery and postoperative period well-prepared. Patients appreciated that the information was provided in print and digital formats, including information videos. An important topic that was missed by many patients was practical information regarding the day of the surgery, such as the time schedule and details about what to expect regarding their physical condition immediately after the surgery and common postoperative discomforts (e.g., nausea, pain, and fatigue). Patients indicated that they would have appreciated receiving this information beforehand, enabling them to mentally prepare and make necessary arrangements at home (Table 3, quote #1).

#### Hospital Admission and Discharge

##### Healthcare Professional-Patient Interaction

Many patients were highly impressed with the healthcare professionals who cared for them during their time in the hospital, offering patients a listening ear and proactively responding to their needs and wishes. This made patients feel that sufficient time was allocated to them, they felt heard and they felt acknowledged (Table 3, quote #2, 3). However, a different experience was also shared by a few patients who conveyed dissatisfaction with the course of events. Their perception was that the nurses on the ward were very busy, which prevented them from doing their job properly. A few patients specifically mentioned that they appreciated that the surgeon visited the ward that afternoon to check in on them.

##### Perspective on Discharge

Upon discharge, the nurses presented patients with information and instructions for the first 24 h at home, including details about medication and remote monitoring. The patient’s informal caregiver participated in the presentation. This was considered beneficial by patients as they were still “groggy” from anesthesia, making it challenging to absorb the information (Table 3, quote #4). Patients’ evaluation of feeling safe to return home was largely based on their current state of physical well-being. Many patients felt relatively well, which contributed to their sense of safety in leaving the hospital (Table 3, quote #5, 6). However, a few patients felt apprehensive about returning home. The risk of postoperative complications led them to doubt the safety of SDD after RYGB. They would have preferred an overnight stay, having all the medical expertise readily available in case of any unforeseen circumstances. While many patients had complete faith in the medical field, stating that if SDD after RYGB was not safe, the hospital would not have offered it, a few patients lacked that faith. It was suggested by one patient that the hospital was primarily concerned with finances, striving to discharge patients as quickly as possible to make room for new admission.

#### Postoperative Trajectory

##### Physical Recovery

Many patients were glad that they were in the SDD care pathway. They preferred to spend the first night after the surgery at home rather than in the hospital and indicated that it was comforting to be in their own familiar environment (Table 3, quote nos. 7 and 8). Nausea was often mentioned by patients. Other postoperative discomforts mentioned by patients were pain and fatigue. Generally, patients were surprised at how quickly they regained their mobility. They felt well enough to walk short distances, both indoors and outdoors, the day after the surgery. Many patients found themselves regaining a state of physical well-being more quickly than they had expected. They mentioned that they no longer needed analgesics shortly after the surgery.

##### Shifted Responsibilities

While some patients appreciated the high level of responsibility that was placed on them within the SDD care pathway, others mentioned that they felt SDD after RYGB places too much responsibility on patients and their caregivers. They emphasized that neither they nor their relatives or friends are medically knowledgeable and questioned whether they are capable of always accurately assessing medical situations and making the right decisions at home (Table 3, quote no. 9 and 10). The presence of healthcare professionals, which gives them a sense of safety and confidence that their condition is closely monitored, was missed by these patients after returning home. Nevertheless, patients emphasized the importance of having a relative or friend with them the first 24 h at home, as they required both physical and mental support. Overall, some patients appreciated the high level of responsibility that was placed on them within the SDD care pathway, while others would have preferred to receive more guidance from healthcare professionals.

##### Remote Monitoring

Generally, patients indicated that they encountered no difficulties with measuring their temperature, pulse, and oxygen saturation. The equipment was considered user-friendly and the instructions on how to respond to abnormal values were clear (Table 3, quote no. 11). Many patients valued vital signs monitoring as it provided them insights about their condition, which served as reassurance (Table 3, quote no. 12). Some patients did not see the added value of this activity. They mentioned that they would have noticed if, for example, they had a fever and would not have needed to measure their temperature for that purpose. For these patients, this activity could have been omitted.

##### Phone Consultation

The phone consultation with the surgeon the day after the surgery was highly appreciated by most patients. They found it very considerate that the surgeon called them. This consultation was viewed as an important part of patient-centered care. Most patients indicated that this consultation should not be omitted and the initiative for this consultation should not be left to patients as patients experience a barrier to reaching out to healthcare professionals when there is no urgency (Table 3, quote nos. 13 and 14).

## Discussion

The aim of this study was to explore patient satisfaction and experience among individuals undergoing laparoscopic Roux-en-Y gastric bypass with SDD. To the best of our knowledge, this study is the first investigation regarding patient satisfaction of RYGB with SDD using a mixed-method approach. The findings from our research suggest that patients are generally satisfied with the care provided, the opportunity to sleep at home, and their fast recovery after RYGB with SDD. This aligns with prior studies where high satisfaction rates were observed following laparoscopic sleeve gastrectomy with SDD [20, 21, 30]. Furthermore, in a feasibility study by Nijland et al., patient satisfaction using a self-developed questionnaire on RYGB with SDD was high, and most patients would recommend this way of treatment to others [11]. Conversely, hospitals that achieve high patient satisfaction also tend to deliver more efficient care, resulting in shorter lengths of stay for surgical patients. These hospitals also have higher surgical process quality, lower surgical readmission rates, and lower surgical mortality rates [31]. Therefore, patient satisfaction is a crucial factor for ensuring the safety and effectiveness of SDD, as it correlates with length of stay.

A noteworthy finding in this study is the satisfaction with information provision, particularly regarding the day of the surgery. This was the only questionnaire that exhibited significant differences between patients who underwent RYGB with SDD and patients with overnight hospitalization after RYGB. The questionnaires revealed significantly lower scores for information provision among patients in the present study. This aspect also stood out in the interviews, where some patients expressed a lack of information regarding the events on the day of surgery. This finding is not unexpected given that SDD represents a new healthcare pathway, and with novelty comes a need for education [32]. Evidently, the current protocol for information provision is not sufficient, a concern we had anticipated for prior to this study. In an effort to address this issue, we developed animation videos about the day of the surgery. Despite their unavailability during this study, we strongly recommend the implementation of some sort of digital information like videos. Effective information provision and expectation management are crucial factors for the success rate and safety of early discharge and SDD [6, 12, 33]. It is important to provide the right information at the right time, so that the information is received when it is needed and in a way that will be the most helpful [34, 35]. Therefore, it is essential to pay careful attention to the information provision and expectation management when implementing a comparable healthcare pathway. For example, preoperative information could include details about common postoperative discomforts and the importance of early mobilization after surgery.

A positive finding in both the quantitative and qualitative part of this study was patient satisfaction with the hospital personnel, including the surgeon, medical team, and office staff. Patients felt seen, heard, and supported. We attribute this outcome mainly to our dedicated bariatric team, which includes specialized nurses and operating room personnel. Patient satisfaction with hospital staff is essential for ensuring high quality care [13]. For example, a prior study introduced a Bariatric Care Coaching Program to improve patient experiences by providing consistent care and communication [36]. This program potentially led to a decrease in avoidable causes of early postoperative readmissions, phone calls, and extended hospital stays [36]. Additionally, in bariatric surgery, trust in the doctor can be regarded as highly important [37]. These outcomes highlight the crucial role of all hospital personnel in providing consistent information to patients, which we believe is an important factor for the success of SDD protocols.

Opinions on the discharge process varied among the patients that were interviewed. Some patients were content with returning home for the comfort of their own bed, while others perceived it as being sent away from the hospital. We emphasize the importance of offering patients the choice between SDD and overnight hospitalization, prioritizing their sense of safety over hospital production. However, in our opinion, this decision should ideally be discussed during the preoperative trajectory to avoid compromising hospital capacity on the day of surgery. Regarding the matter of shifted responsibilities, opinions varied as well. Patients and their caregivers were in control of identifying potential complications. Some found this arrangement reassuring and convenient, while others felt insecure and incapable of bearing such responsibility. Based on our clinical experience, individual variations on this matter were expected. This underscores the need for patients to have the option to decline SDD if they feel uncomfortable with the shifted responsibilities. However, it is important to keep in mind that the healthcare system currently faces significant challenges, particularly in managing hospital capacity and staff [38]. While patient satisfaction plays an important role in quality of care, we deem it vital to balance this with healthcare recourse utilization. Naturally, this is only possible if medical safety remains guaranteed and the SDD protocol can be responsibly provided. The establishment of a safety net remains crucial for monitoring potential complications in bariatric surgery with SDD, as valued by patients and vital for healthcare providers [7].

The mixed-methods approach was a strength of this study. Combination of quantitative and qualitative data helped us to provide and in-depth understanding of patient satisfaction and experience with SDD after RYGB. Researchers from different fields and disciplines with extensive experience with both types of research were involved in this study, strengthening the interpretation of the results. Furthermore, our study used the validated BODY-Q and compared its data with that of a control group. Two systematic reviews assessing Patient-Reported Outcome Measures (PROMs) in bariatric surgery have highlighted the BODY-Q as the most rigorously developed and validated PROM for this population [39, 40]. However, this study has several limitations as well. There were significant baseline differences between the control group and the group in the present study. This was expected since the control group represented the general bariatric population, while the SDD group consisted only of patients with a low risk of complications, resulting in a younger population with fewer comorbidities. This difference in baseline characteristics could have introduced bias into the study. Ideally, comparing two groups with similar characteristics would have been preferable. However, due to the established implementation of SDD in our hospital, this was not a feasible option. Another limitation could be a potential selection bias regarding the questionnaires. The response rate for completing the questionnaires was 76%, which is satisfactory and above the average for surgical survey responses [41]. However, the thoughts of the 16 patients on SDD remain unknown as they did not respond. Furthermore, the quantitative sample size was limited to 51 questionnaires, which may restrict generalization due to the specific nature of this pathway. Nevertheless, the insights gained from this study could hold relevance for professionals aiming to implement similar pathways.

## Conclusion

Patient satisfaction with SDD after gastric bypass surgery is high. While not every patient might be suitable for this healthcare pathway, offering the choice between SDD and overnight hospitalization remains crucial, irrespective of meeting selection criteria. Patients should feel confident about going home on the same day as the surgery, as the responsibility for monitoring potential complications shifts from the healthcare provider to the patient and their informal caregivers. Moreover, special attention should be given to providing information, particularly concerning the day of the surgery.

## Data Availability

The data supporting the findings of this study are available upon request from the corresponding author.

## Appendix

Table 4

**Table 4:** Topic list interviews

Start interview
• You recently underwent bariatric surgery in OLVG hospital. After the surgery, you didn’t need to stay overnight in the hospital; instead, you went home the same day. I’m curious about your experience with this, and I’d like to discuss it with you during this interview. The surgery took place … days ago. How are you feeling now?
Preoperative
• What were the reasons for choosing OLVG hospital for you? • Most Dutch centers do not perform this operation with same-day discharge. What is your opinion about OLVG hospital performing this operation with same-day discharge? Could you elaborate on this? • Did you make the choice for same-day discharge yourself? If yes: why did you choose this? If no: who made this choice? Were you in agreement with it? Why or why not? What were the anticipated advantages of same-day discharge for you? What were the anticipated disadvantages of same-day discharge for you? • What do you think about the information regarding day treatment that you received beforehand? Who provided this information? How was this information given? To what extent was this information clear to you? To what extent was this information sufficient for you? Do you have any suggestions for improvement?
Discharge
• Could you share your experience of the care in the hospital immediately after the operation? What did you find positive about it? What did you find challenging about it? • What is your opinion about the advice and instructions – for instance, regarding pain management – given before you left for home? To what extent was this clear to you? To what extent was this sufficient for you? Do you have any suggestions for improvement? • How safe did you feel about going home? What factors contributed to you feeling safe/unsafe about going home?
Postoperative
• Could you tell us about how you felt in the first few hours after arriving home? • How did the self-monitoring of your heart rate, oxygen saturation, and temperature go after the operation? To what extent was it clear to you how the equipment worked? To what extent was it clear to you what to do in case of abnormal readings? • What were your thoughts on self-monitoring your heart rate, oxygen saturation, and temperature after the operation? To what extent did you find the self-monitoring of your vital signs after the operation beneficial? Or, what did this provide you with? • Research indicates that complications might not be noticed earlier because someone is self-monitoring their heart rate, oxygen saturation, and temperature after the operation. Suppose you hadn’t done this. How does that make you feel? Could you elaborate on this? • How beneficial did you find the surgeon’s phone call the day after the operation? Or, what did this provide you with? Could you elaborate on this? Do you think this could be omitted? Why or why not? How would you feel if this call wasn’t scheduled by default, but instead, you had to initiate contact if needed? • Overall, how satisfied are you with the care provided by OLVG hospital? Could you elaborate on this? Would you recommend day treatment to others? Why or why not? What do you see as the biggest advantages of same-day discharge? What do you see as the biggest disadvantages of same-day discharge? Do you have any suggestions for improvement?
